# A dataset of oracle characters for benchmarking machine learning algorithms

**DOI:** 10.1038/s41597-024-02933-w

**Published:** 2024-01-18

**Authors:** Mei Wang, Weihong Deng

**Affiliations:** School of Artificial Intelligence, Beijing University of Posts and Telecommunications, Beijing, 100876 China

**Keywords:** Electrical and electronic engineering, Imaging and sensing

## Abstract

Oracle bone script is an ancient Chinese writing system engraved on turtle shells and animal bones, serving as a valuable resource for interpreting ancient culture, history, and language. We introduce the Oracle-MNIST dataset, comprising of 28 × 28 grayscale images of 30,222 ancient characters from 10 categories, designed for benchmarking pattern classification, with particular challenges related to image noise and distortion. The training set totally consists of 27,222 images, and the test set contains 300 images per class. Oracle-MNIST follows the same data format with the original MNIST dataset, enabling direct compatibility with all existing classifiers and systems, but it constitutes a more challenging classification task than MNIST. The images of ancient characters suffer from (1) extremely serious and unique noises caused by three-thousand years of burial and aging and (2) dramatically variant writing styles by ancient Chinese, which all make them realistic for machine learning research.

## Background & Summary

In the last few years, rapid progress has been unfolding in machine learning (ML) due to the release of specialized datasets that serve as experimental testbeds and public benchmarks, thus focusing the efforts of the research community. The most widely known dataset in computer vision is the MNIST dataset, which was first introduced in 1998 by Lecun *et al*.^1^. MNIST is a 10-class digit classification dataset, and consists of 60,000 grayscale images for training and 10,000 grayscale images for testing. The entire dataset is relatively small, free to access and use, and is encoded and stored in an entirely straightforward manner, which have almost certainly contributed to its widespread use.

However, with the discovery of improved learning algorithms, the performance has been saturated on MNIST. For example, Convolutional Neural Networks (CNNs)^2,3^ can easily achieve an accuracy of above 99%. *This is partially attributed to the benchmark that does not capture requirements of many real-world scenarios*. To avoid the saturated performance and offer challenges for the improved ML algorithms, some modified MNIST datasets are constructed, e.g., EMNIST^4^ and Fashion-MNIST^5^. EMNIST extends the number of classes by introducing uppercase and lowercase letters, but the extra classes require a change of the framework of deep neural network used by MNIST. Fashion-MNIST contains 70,000 grayscale images of 10-class fashion products. These product images are taken from Zalando’s website, which is the Europe’s largest online fashion platform (http://www.zalando.com). They are shot by professional photographers, and thus are clear and standardized. However, it fails to capture as wide of a range of variations as possible in the real world.

The purpose of this paper is to provide a realistic and challenging dataset, called Oracle-MNIST, to facilitate easy and fast evaluation for ML algorithms on the real-world images of ancient characters. Oracle-MNIST contains 30,222 images of oracle characters belonging to 10 categories.

- **Real-world challenge**. Different from handwritten digits, oracle characters are scanned from the real oracle-bone surface. Therefore, Oracle-MNIST suffers from extremely serious and unique noises caused by thousands of years of burial and aging, and contains various writing styles in each category, all of which make it more realistic and difficult for ML research.
- **Ease-of-use**. Following the original MNIST, the images in Oracle-MNIST have 28 × 28 grayscale pixels. It can be immediately compatible with any ML package capable of working with the MNIST dataset since it shares the same data format. In fact, the only change one needs to make to use this dataset is to change the URL from where the MNIST dataset is fetched.

We introduce this dataset specifically made for machine learning research to serve as a direct drop-in replacement for the original MNIST dataset and engage the community to the field of Chinese ancient literature, which contributes not only to technology but also to culture heritage preservation and the understanding of oracle characters and ancient civilization.

## Methods

### Discovery of oracle characters

**Fig. 1:**
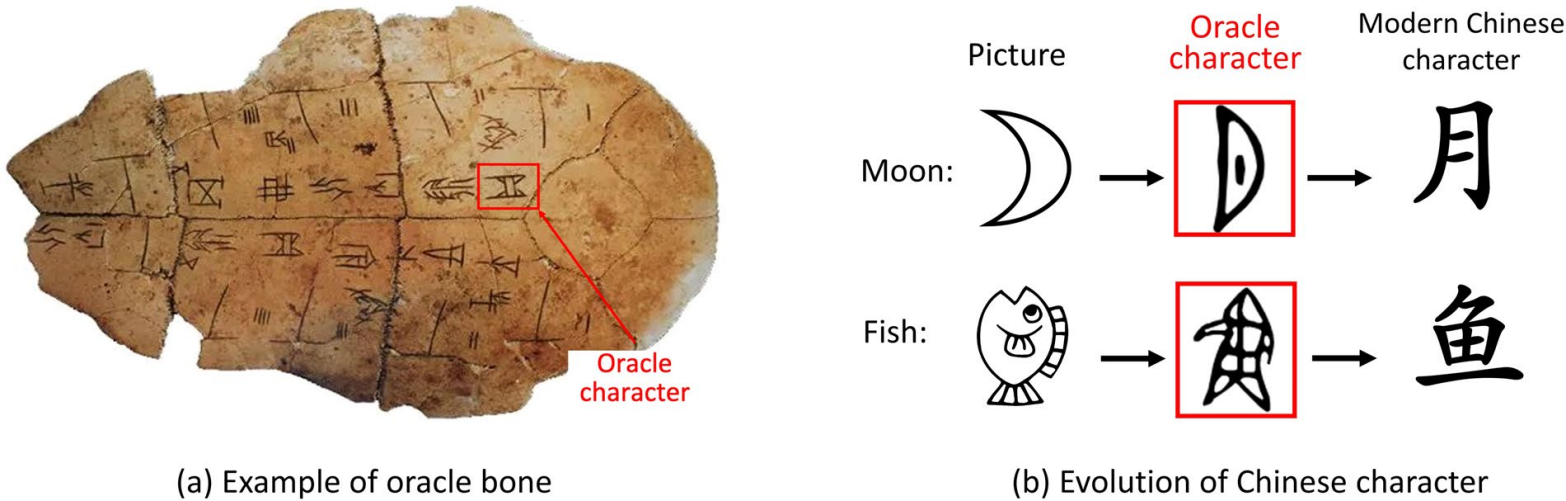
Oracle characters are the oldest hieroglyphs in China, which were inscribed on (**a**) oracle bones about 3000 years ago. (**b**) Despite the pictorial nature of oracle characters, they constitute a fully functional and well-developed writing system.

Ancient history relies on the study of ancient characters. As the oldest hieroglyphs in China, oracle characters^6,7^, with a history spanning nearly three millennia, have contributed greatly to modern civilization, enabling the Chinese culture to be passed on from generation to generation and become the only civilization to last up to the present. As shown in Fig. 1, oracle characters are engraved on tortoise shells and animal bones, and record the life and history of the Shang Dynasty (around 1600-1046 B.C.), including divination practices, war expeditions, hunting, medical treatments, and childbirth. They were first discovered by a merchant called Wang Xirong in 1899, during the Qing Dynasty (1644–1911). In the early 20th century, Chinese researchers excavated numerous oracle bones at Xiaotun Village in Anyang, Henan Province, capital of the Shang Dynasty. Since then, the research on oracle characters has attracted much attention. It is of vital importance for Chinese etymologies and calligraphy as well as for learning the culture and history of ancient China and even the world.

**Fig. 2:**
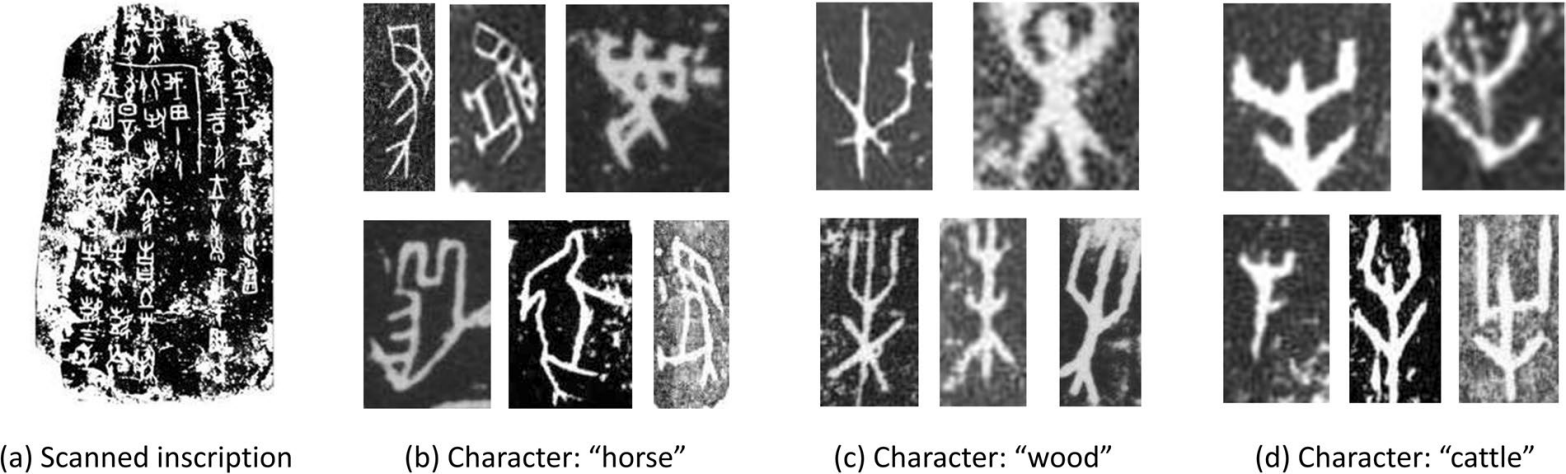
(**a**) Example of scanned oracle inscription. (**b**–**d**) Examples of scanned oracle characters. Different writing styles lead to a high degree of intra-class variance and inter-class similarity.

Most of oracle characters are stored by scanned images, which are generated by reproducing the oracle-bone surface by placing a piece of paper over the subject and then rubbing the paper with rolled ink, as shown in Fig. 2a. Recognizing these oracle characters is difficult for both experts and machines. Thus far, nearly 4,500 different oracle characters have been discovered, but only about 2,200 characters have been successfully deciphered. The reasons are as follows. (1) **Abrasion and noise**. Many oracle-bone inscriptions have been damaged over the centuries and their texts are now fragmentary. The aging process has also made the inscriptions less legible, resulting in broken characters with serious noise. (2) **Large variance**. Different writing styles lead to a high degree of intra-class variance. Characters belonging to the same category largely vary in stroke and even topology, as shown in Fig. 2b. Some characters belonging to different categories are similar to each other, which brings great difficulty for recognition. For example, the characters of ‘wood’ and ‘cattle’ categories only differ in some small details shown in Fig. 2c,d. Clearly, providing such Oracle-MNIST benchmark for ML community would facilitate research towards oracle character recognition and help to handle these challenges from the perspective of computer vision. We also hope that archaeologists and paleographists can gain from the progress achieved by ML community in the future such that their workload can be lightened when identifying characters.

### Construction of oracle-MNIST

Oracle-MNIST is based on the collection of YinQiWenYuan website (http://jgw.aynu.edu.cn/ajaxpage/home2.0). It is a large oracle-bone platform constructed by AnYang Normal University. The raw images of oracle characters are collected from eight authoritative oracle-bone publications, e.g., Jiaguwen heji^8^. Then, oracle characters are cropped from these raw images such that each cropped image is centered by one single character. Most of the per-character images have gray or black backgrounds and vary in resolution. Since these oracle characters are scanned from the real oracle-bone surface, they are broken and suffer from serious noises. The meanings of characters are utilized as their class labels. The labels are manually annotated by experts in archeology or paleography.

**Fig. 3:**
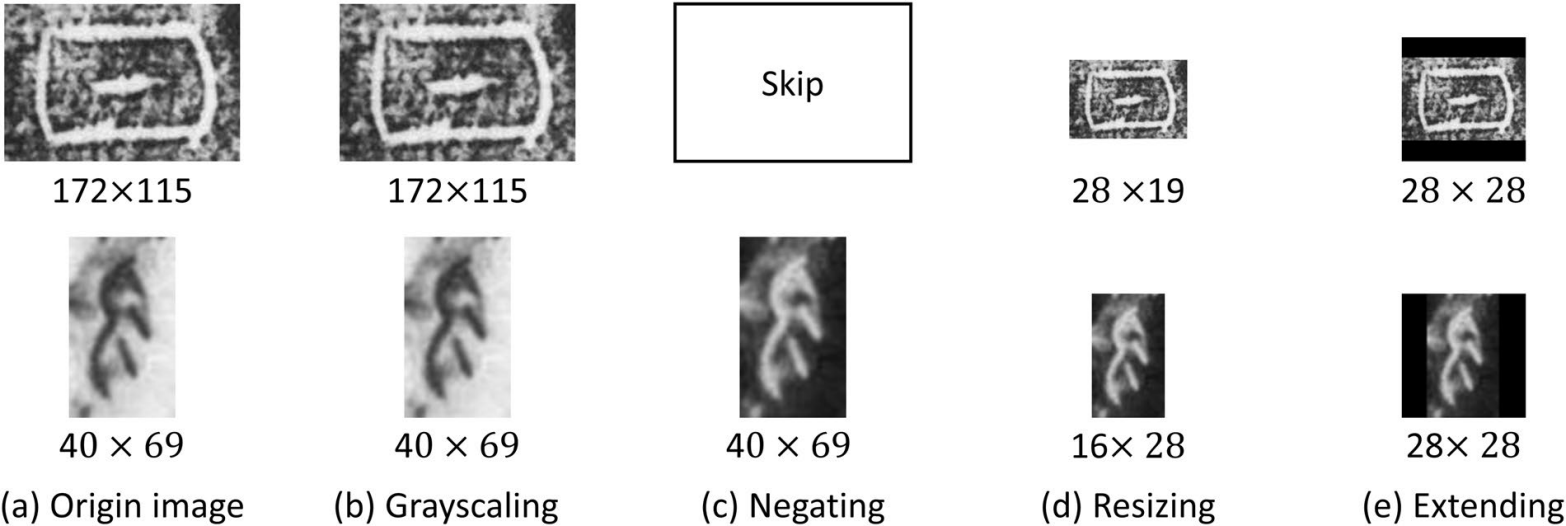
Diagram of the conversion process used to generate Oracle-MNIST dataset. Two examples from ‘sun’ and ‘not’ categories are depicted, respectively.

To build Oracle-MNIST, we selected 30,222 commonly-used characters of 10 classes. The selected images are then fed into the following conversion pipeline such that they can be converted to 28 × 28 pixel 8-bit grayscale images that match the characteristics of the digits in the MNIST dataset. An overview of the conversion process is visualized in Fig. 3.

- **Grayscaling**. The original RGB images are converted to 8-bit grayscale pixels as shown in Fig. 3b.
- **Negating**. Most of these scanned images contain white characters on black backgrounds; and conversely, a few images consist of black characters on white backgrounds. For consistency, we negate the intensities of the image if its foreground is darker than the background shown in Fig. 3c. The negating process can be performed by: *p*_*new*_ = 255−*p*_*old*_, where *p*_*old*_ and *p*_*new*_ are the intensity values of images before and after negating.
- **Resizing**. With its aspect ratio preserved, the longest edge of the image is resized to 28 using a bi-cubic interpolation algorithm, as shown in Fig. 3d.
- **Extending**. We extend the shortest edge to 28 by padding it with 0, and put the image to the center of the canvas. The range of intensity values is then scaled to [0, 255], resulting in the 28 × 28 pixel grayscale images shown in Fig. 3e.

We also attempt to process the images by some image enhancement techniques, e.g., gray stretch and histogram equalization. Although the visual quality of images is successfully improved, the recognition performance would slightly degrade. Therefore, no image enhancement technology is applied to Oracle-MNIST. We also make the original RGB images available and left the data processing job to the algorithm developers.

We chose to resize the images to a resolution of 28 × 28 to follow the same data format as the original MNIST dataset, ensuring direct compatibility with all existing classifiers and systems. However, considering that today’s hardware allows for deep learning to operate on a larger scale, we also provide a version with a resolution of 224 × 224.

## Data Records

**Fig. 4:**
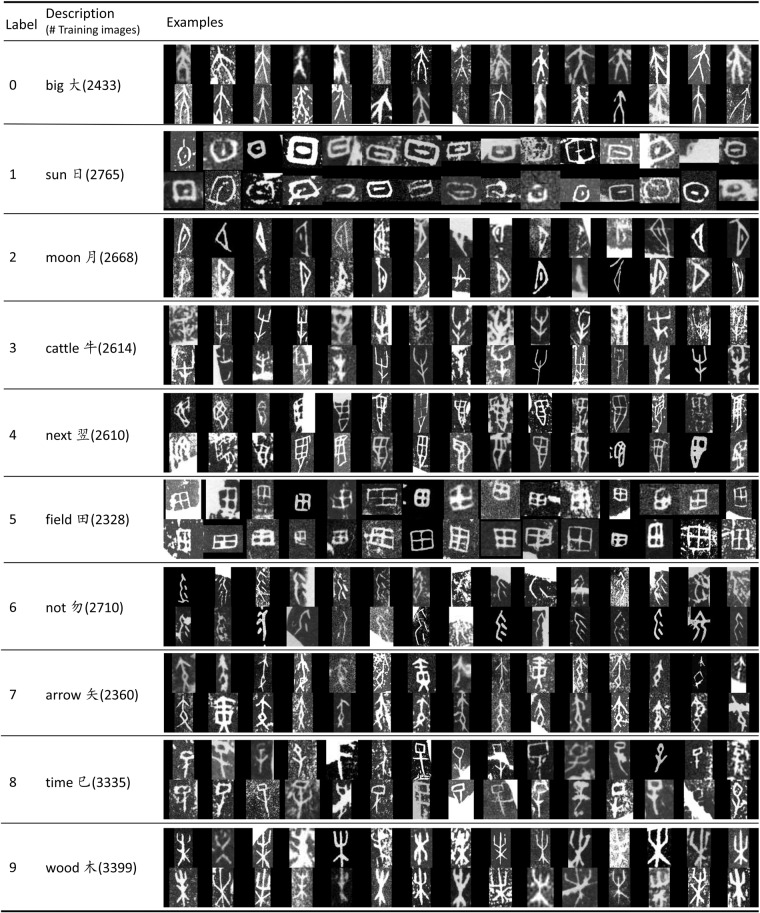
Class labels, example images and the number of training images in Oracle-MNIST dataset. “time UTF8gkai” represents 9–11 a.m. (one of the Earthly Branches which are ancient China’s systems for keeping time).

Oracle-MNIST dataset contains 30,222 samples of 10 classes, where each class represents a unique oracle bone glyph character. Figure 4 gives a summary of all class labels in Oracle-MNIST with examples for each class. The dataset is divided into a training and a test set, and we make sure that they are disjoint. The training set totally consists of randomly-selected 27,222 images belonging to 10 categories. It is class-imbalanced due to the appearance frequency in the real source books, ranging from 3,399 examples to 2,328 examples per class. The test set contains 10 classes with 300 images per class.

Oracle-MNIST can be accessed at Figshare^9^, Science Data Bank^10^, GitHub (https://github.com/wm-bupt/oracle-mnist). We grant free access to the dataset, without the need for user registration. The dataset is distributed in GZIP archives with a total size of 13.8 MBytes. Images and labels are stored in the same IDX file format as the MNIST dataset, which is designed for storing vectors and multidimensional matrices. The result files are listed in Table 1. Although our test set consists of only 3 K images, it is called ‘t**10**k’ instead of ‘t**3**k’ to be consistent with the original MNIST dataset such that it can be easily compatible with any ML package.

**Table 1:** Files contained in the Oracle-MNIST dataset.

Name	Description	#Images	# Classes	Size
train-images-idx3-ubyte.gz	Training set images	27,222	10	12.4 MBytes
train-labels-idx1-ubyte.gz	Training set labels	27,222	10	13.7 KBytes
t10k-images-idx3-ubyte.gz	Test set images	3,000	10	1.4 MBytes
t10k-labels-idx1-ubyte.gz	Test set labels	3,000	10	1.6 KBytes

The images with resolution of 224 × 224 are also available at Figshare^9^ and Science Data Bank^10^. The original RBG images can be downloaded from GitHub. These images are also split into a training and a test set. All of the images in BMP format are grouped by folders with labels from 0–9 representing the class labels. The images in the same folder belong to the same category. Each image is named as ‘******_#.bmp’, where ‘******’ represents the class labels (6-digit code) provided by YinQiWenYuan website and ‘#’ represents the image name.

## Technical Validation

We evaluate some algorithms with different parameters on Oracle-MNIST and report the results in Tables 2–10. For each algorithm, the average classification accuracy is reported based on three repeated experiments. The benchmarks on the MNIST and Fashion-MNIST dataset are also included for a side-by-side comparison.

**Table 2:** Benchmark results using CNN on Oracle-MNIST, Fashion-MNIST and MNIST.

Parameter	Oracle (%)	Fashion (%)	MNIST (%)
2 × Conv-Pool-ReLu, 2 × FC, Dropout	93.8	92.1	99.3
2 × Conv-Pool-ReLu, 2 × FC	92.8	90.8	99.4
1 × Conv-Pool-ReLu, 2 × FC	91.6	91.2	99.2

**Table 3:** Benchmark results using GradientBoostingClassifier on Oracle-MNIST, Fashion-MNIST and MNIST.

Parameter	Oracle (%)	Fashion (%)	MNIST (%)
n_estimators = 100, loss = deviance, max_depth = 10	72.5	88.0	96.9
n_estimators = 50, loss = deviance, max_depth = 10	69.9	87.2	96.4
n_estimators = 100, loss = deviance, max_depth = 3	69.7	86.2	94.9
n_estimators = 50, loss = deviance, max_depth = 3	64.6	84.0	92.6
n_estimators = 10, loss = deviance, max_depth = 10	59.9	84.9	93.3

**Table 4:** Benchmark results using SVC on Oracle-MNIST, Fashion-MNIST and MNIST.

Parameter	Oracle (%)	Fashion (%)	MNIST (%)
C = 10, kernel = rbf	75.5	89.7	97.3
C = 100, kernel = rbf	75.0	89.0	97.2
C = 100, kernel = poly	74.5	89.0	97.8
C = 10, kernel = poly	73.2	89.1	97.6
C = 1, kernel = rbf	71.3	87.9	96.6
C = 1, kernel = poly	62.9	87.3	95.7
C = 1, kernel = linear	57.6	83.9	92.9
C = 10, kernel = linear	56.7	82.9	92.7
C = 100, kernel = linear	56.2	82.7	92.6

**Table 5:** Benchmark results using MLPClassifier on Oracle-MNIST, Fashion-MNIST and MNIST.

Parameter	Oracle (%)	Fashion (%)	MNIST (%)
activation = relu, hidden_layer_sizes = [100]	74.7	87.1	97.2
activation = relu, hidden_layer_sizes = [100, 10]	72.6	87.0	97.2
activation = tanh, hidden_layer_sizes = [100]	66.7	86.8	96.2
activation = tanh, hidden_layer_sizes = [100, 10]	65.5	86.3	95.7
activation = relu, hidden_layer_sizes = [10, 10]	61.2	85.0	93.6
activation = relu, hidden_layer_sizes = [10]	60.7	84.8	93.3
activation = tanh, hidden_layer_sizes = [10]	58.4	84.1	92.1
activation = tanh, hidden_layer_sizes = [10, 10]	58.4	84.0	92.1

**Table 6:** Benchmark results using RandomForestClassifier on Oracle-MNIST, Fashion-MNIST and MNIST.

Parameter	Oracle (%)	Fashion (%)	MNIST (%)
n_estimators = 100, criterion = gini, max_depth = 100	65.0	87.2	97.0
n_estimators = 100, criterion = entropy, max_depth = 50	65.0	87.2	96.9
n_estimators = 100, criterion = gini, max_depth = 50	64.9	87.1	97.1
n_estimators = 100, criterion = entropy, max_depth = 100	64.9	87.3	97.0
n_estimators = 50, criterion = gini, max_depth = 100	63.6	86.9	96.7
n_estimators = 50, criterion = entropy, max_depth = 50	62.9	87.1	96.7
n_estimators = 50, criterion = gini, max_depth = 50	62.6	87.0	96.8
n_estimators = 50, criterion = entropy, max_depth = 100	62.5	87.2	96.8
n_estimators = 100, criterion = gini, max_depth = 10	58.3	83.5	94.9
n_estimators = 100, criterion = entropy, max_depth = 10	58.3	83.8	95.0
n_estimators = 50, criterion = entropy, max_depth = 10	58.0	83.8	94.7
n_estimators = 50, criterion = gini, max_depth = 10	57.6	83.4	94.5
n_estimators = 10, criterion = gini, max_depth = 10	53.2	82.5	93.0
n_estimators = 10, criterion = entropy, max_depth = 10	52.8	82.8	93.3
n_estimators = 10, criterion = entropy, max_depth = 100	52.1	85.2	94.9
n_estimators = 10, criterion = gini, max_depth = 100	52.0	84.7	94.8
n_estimators = 10, criterion = entropy, max_depth = 50	51.8	85.3	94.9
n_estimators = 10, criterion = gini, max_depth = 50	51.3	84.8	94.8

**Table 7:** Benchmark results using KNeighborsClassifier on Oracle-MNIST, Fashion-MNIST and MNIST.

Parameter	Oracle (%)	Fashion (%)	MNIST (%)
weights = distance, n_neighbors = 9, p = 2	62.7	84.9	94.4
weights = distance, n_neighbors = 9, p = 1	61.8	85.4	95.5
weights = uniform, n_neighbors = 9, p = 1	61.6	85.3	95.5
weights = uniform, n_neighbors = 9, p = 2	61.5	84.7	94.3
weights = distance, n_neighbors = 5, p = 1	60.3	85.4	95.9
weights = uniform, n_neighbors = 5, p = 1	59.6	85.2	95.7
weights = distance, n_neighbors = 5, p = 2	59.5	85.2	94.5
weights = uniform, n_neighbors = 5, p = 2	59.0	84.9	94.4
weights = uniform, n_neighbors = 1, p = 1	55.8	83.8	95.5
weights = distance, n_neighbors = 1, p = 1	55.8	83.8	95.5
weights = distance, n_neighbors = 1, p = 2	55.7	83.9	94.3
weights = uniform, n_neighbors = 1, p = 2	55.7	83.9	94.3

**Table 8:** Benchmark results using LogisticRegression on Oracle-MNIST, Fashion-MNIST and MNIST.

Parameter	Oracle (%)	Fashion (%)	MNIST (%)
C = 10, multi_class = ovr, penalty = l2	59.8	83.9	91.6
C = 100, multi_class = ovr, penalty = l2	59.8	83.6	91.6
C = 1, multi_class = ovr, penalty = l2	59.7	84.1	91.7

**Table 9:** Benchmark results using SGDClassifier on Oracle-MNIST, Fashion-MNIST and MNIST.

Parameter	Oracle (%)	Fashion (%)	MNIST (%)
loss = log, penalty = l1	56.7	81.5	91.0
loss = log, penalty = elasticnet	56.0	81.6	91.2
loss = hinge, penalty = l1	55.6	81.5	91.1
loss = log, penalty = l2	55.1	81.3	91.3
loss = hinge, penalty = l2	54.9	81.9	91.4
loss = hinge, penalty = elasticnet	54.7	81.6	91.3
loss = modified_huber, penalty = elasticnet	51.5	81.3	91.4
loss = modified_huber, penalty = l1	50.7	81.7	91.0
loss = perceptron, penalty = l1	49.4	81.8	91.2
loss = modified_huber, penalty = l2	49.0	81.6	91.3
loss = perceptron, penalty = l2	47.9	81.4	91.3
loss = perceptron, penalty = elasticnet	47.4	81.4	91.2
loss = squared_hinge, penalty = l1	46.5	81.3	91.1
loss = squared_hinge, penalty = l2	45.0	81.4	91.2
loss = squared_hinge, penalty = elasticnet	42.1	81.5	91.4

**Table 10:** Benchmark results using LinearSVC on Oracle-MNIST, Fashion-MNIST and MNIST.

Parameter	Oracle (%)	Fashion (%)	MNIST (%)
loss = hinge, C = 1, multi_class = crammer_singer, penalty = l2	58.1	83.5	91.9
loss = squared_hinge, C = 1, multi_class = crammer_singer, penalty = l2	58.0	83.4	91.9
loss = hinge, C = 1, multi_class = crammer_singer, penalty = l1	57.8	83.3	91.9
loss = squared_hinge, C = 1, multi_class = crammer_singer, penalty = l1	57.3	83.3	91.9
loss = squared_hinge, C = 1, multi_class = ovr, penalty = l2	55.8	82.0	91.2
loss = hinge, C = 1, multi_class = ovr, penalty = l2	54.8	83.6	91.7

From the results, we have the following observations. First, classic (shallow) ML algorithms can easily achieve 97% on the MNIST dataset which proves that MNIST is too easy to evaluate the algorithms. Our Oracle-MNIST dataset provides 10-class images of ancient characters and further captures as wide of a range of variations as possible in the real world to pose a more challenging classification task than the MNIST digits data and Fashion-MNIST data. As we can see that all classic (shallow) ML algorithms perform the best on MNIST, followed by Fashion-MNIST, and the worst on Oracle-MNIST. For example, the random forest classifier achieves the accuracies of 97.1%, 87.1% and 64.9%, respectively. This is because a high degree of intra-class variance and inter-class similarity as we described above would bring great difficulty for classification. Moreover, the scanned oracle images are seriously degraded and even completely lost their discriminative glyph information caused by blur, noise and occlusion.

Second, CNN outperforms all of the classic (shallow) ML algorithms on Oracle-MNIST. Benefitting from local receptive fields and spatial or temporal subsampling, CNN can force the extraction of local features and reduce the sensitivity of the output to shifts and distortions^11^. As a result, real-world challenges such as different writing styles, noise, and occlusion can be tackled to some extent, leading to better performance on oracle characters^12^. However, the performance on Oracle-MNIST has not been saturated. The CNN utilized in this paper achieves an error rate of 6.2% on Oracle-MNIST, and there is still room for improvement. Despite the powerful representation ability of CNN, the problem of recognizing these ancient characters remains to be fully solved.

## Usage Notes

We provide a Python script *mnist_reader.py* that can be used to read the images and labels from the files of our Oracle-MNIST. It is provided together with the database on GitHub. Since Oracle-MNIST is converted to a format that is directly compatible with classifiers built to handle the MNIST dataset, the only change one needs to make to use this dataset is to change the URL from where the MNIST dataset is fetched. We also provide a Python script *train_pytorch.py* to enable researches to reproduce the results of CNNs utilized in this paper.

## Data Availability

Oracle-MNIST are freely available online at GitHub (https://github.com/wm-bupt/oracle-mnist). Tutorials for loading the dataset and code for training and testing oracle character recognition models are also publicly available without restriction.
